# Isotopic signature and nano-texture of cesium-rich micro-particles: Release of uranium and fission products from the Fukushima Daiichi Nuclear Power Plant

**DOI:** 10.1038/s41598-017-05910-z

**Published:** 2017-07-14

**Authors:** Junpei Imoto, Asumi Ochiai, Genki Furuki, Mizuki Suetake, Ryohei Ikehara, Kenji Horie, Mami Takehara, Shinya Yamasaki, Kenji Nanba, Toshihiko Ohnuki, Gareth T. W. Law, Bernd Grambow, Rodney C. Ewing, Satoshi Utsunomiya

**Affiliations:** 1 Department of Chemistry, Kyushu University, 744 Motooka, Nishi-ku, Fukuoka-shi, Fukuoka, 819-0395 Japan; 2 National Institute of Polar Research, 10-3, Midori-cho, Tachikawa-shi, Tokyo, 190-8518 Japan; 3 Department of Polar Science, The Graduate University for Advanced Studies (SOKENDAI), Shonan Village, Hayama, Kanagawa 240-0193 Japan; 4 Faculty of Pure and Applied Sciences and Center for Research in Isotopes and Environmental Dynamics, University of Tsukuba, 1-1-1 Tennodai, Tsukuba, Ibaraki, 305-8577 Japan; 5 Department of Environment System Management, Faculty of Symbiotic Systems Science, Fukushima University, 1 Kanayagawa, Fukushima, 960-1296 Japan; 6 Laboratory for Advanced Nuclear Energy, Institute of Innovative Research, Tokyo Institute of Technology, 2-12-1 Ookayama, Meguro-ku, Tokyo, 152-8550 Japan; 7 Centre for Radiochemistry Research, School of Chemistry, The University of Manchester, Oxford Road, Manchester, M13 9PL UK; 8 SUBATECH, IMT Atlantique, the University of Nantes CNRS/IN2P3, Nantes, 44307 France; 9 Department of Geological Sciences and Center for International Security and Cooperation, Stanford University, Stanford, CA 94305-2115 USA

## Abstract

Highly radioactive cesium-rich microparticles (CsMPs) released from the Fukushima Daiichi Nuclear Power Plant (FDNPP) provide nano-scale chemical fingerprints of the 2011 tragedy. U, Cs, Ba, Rb, K, and Ca isotopic ratios were determined on three CsMPs (3.79–780 Bq) collected within ~10 km from the FDNPP to determine the CsMPs’ origin and mechanism of formation. Apart from crystalline Fe-pollucite, CsFeSi_2_O_6_ · nH_2_O, CsMPs are comprised mainly of Zn–Fe-oxide nanoparticles in a SiO_2_ glass matrix (up to ~30 wt% of Cs and ~1 wt% of U mainly associated with Zn–Fe-oxide). The ^235^U/^238^U values in two CsMPs: 0.030 (±0.005) and 0.029 (±0.003), are consistent with that of enriched nuclear fuel. The values are higher than the average burnup estimated by the ORIGEN code and lower than non-irradiated fuel, suggesting non-uniform volatilization of U from melted fuels with different levels of burnup, followed by sorption onto Zn–Fe-oxides. The nano-scale texture and isotopic analyses provide a partial record of the chemical reactions that occurred in the fuel during meltdown. Also, the CsMPs were an important medium of transport for the released radionuclides in a respirable form.

## Introduction

Radionuclides with ~520 PBq initial total activity were released from the Fukushima Daiichi Nuclear Power Plant (FDNPP) as a result of the nuclear disaster that occurred after the Tohoku earthquake on March 11, 2011^1^. The released radionuclides, including noble gases (Xe and Kr) and volatile fission products (I, Cs, Te, Sb, and Ag), contaminated the surface over ~14,000 km^2^ surrounding the FDNPP^1–5^, requiring the evacuation of some 100,000 residents. Radioactive Cs, ^134^Cs and ^137^Cs, are the most important radionuclides contributing to the high radiation in the environment near the FDNPP at present because of their relatively short half-lives, 2.06 and 30.07 years, respectively^6^. About 1–7% of the Cs inventory of three reactor cores was released^3, 7^. Previous studies of the distribution and migration of radioactive Cs in the surface environment around Fukushima^4–6, 8–12^ indicated that an initially soluble form of radioactive Cs was released from the damaged reactors and spread over the Fukushima Prefecture and the surrounding area through dry and wet deposition^3–5, 8^. Subsequently, a soluble form of Cs was tightly bound to the interlayers of clays, such as vermiculite, and remained within the top ~5 cm of the soil^3, 9, 10^. However, Cs in the contaminated soils is heterogeneous and concentrated locally at the micron scale as hot spots, as revealed through autoradiography^9–11^, and this heterogeneity has not been fully described. A possible cause of the heterogeneity is the formation of Cs-rich microparticles (CsMPs), with a high Cs radioactivity per unit mass, found at a range of distances from the FDNPP^12^. This is another important route of Cs migration in the environments. Different from the soluble Cs, the CsMPs are sparingly soluble in water. The ^134^Cs/^137^Cs radioactivity ratio of ~1 indicates that they originated from the FDNPP. CsMPs were initially considered as amorphous glass particles containing various elements derived from the reactors that were melted into the glass matrix^12–14^. However, recent studies have shown that the Cs concentrations are associated with discrete Zn–Fe-oxide nanoparticles embedded in a pure SiO_2_ glass matrix, as well as numerous nano-scale inclusions with a variety of fission products^15, 16^. The nano-texture within the CsMPs records the chemical reactions that took place during the meltdowns inside the reactors. The sparingly soluble CsMPs were identified as dominant Cs carriers during the initial Cs fallout in Tokyo, Japan, and they are expected to differ from soluble Cs in terms of environmental mobility and health impacts^15^. In addition to the reaction sequence of the CsMPs formation inside the reactors, trace amounts of U are present in CsMPs. The origin of U and the other nuclides remains uncertain, as insulating material in the reactors also contains trace amount of natural U. The occurrence of U derived from inside the reactors is potentially useful for understanding the reactions that nuclear fuels experienced during meltdown and even the status of the melted nuclear fuels in the damaged reactors^17^. This is crucial for developing an appropriate decommissioning strategy. The hypothesis of this study is that the isotopic ratio analysis in combination with atomic-resolution analysis reveals the source of U and the release processes associated with CsMPs from the FDNPP. The CsMPs are a very unique form of condensed matter that was created during the meltdown events inside the reactors at the FDNPP, very different from general concept of Cs release as a soluble Cs species, such as CsI and CsOH. Isotopic analyses of the individual CsMPs are reported for the first time. Isotopic analysis of the other nuclides, stable and radiogenic, also allow us to identify their sources, natural or fissionogenic.

## Results

### Shape, composition, and radioactivity of CsMPs

**Figure 1:**
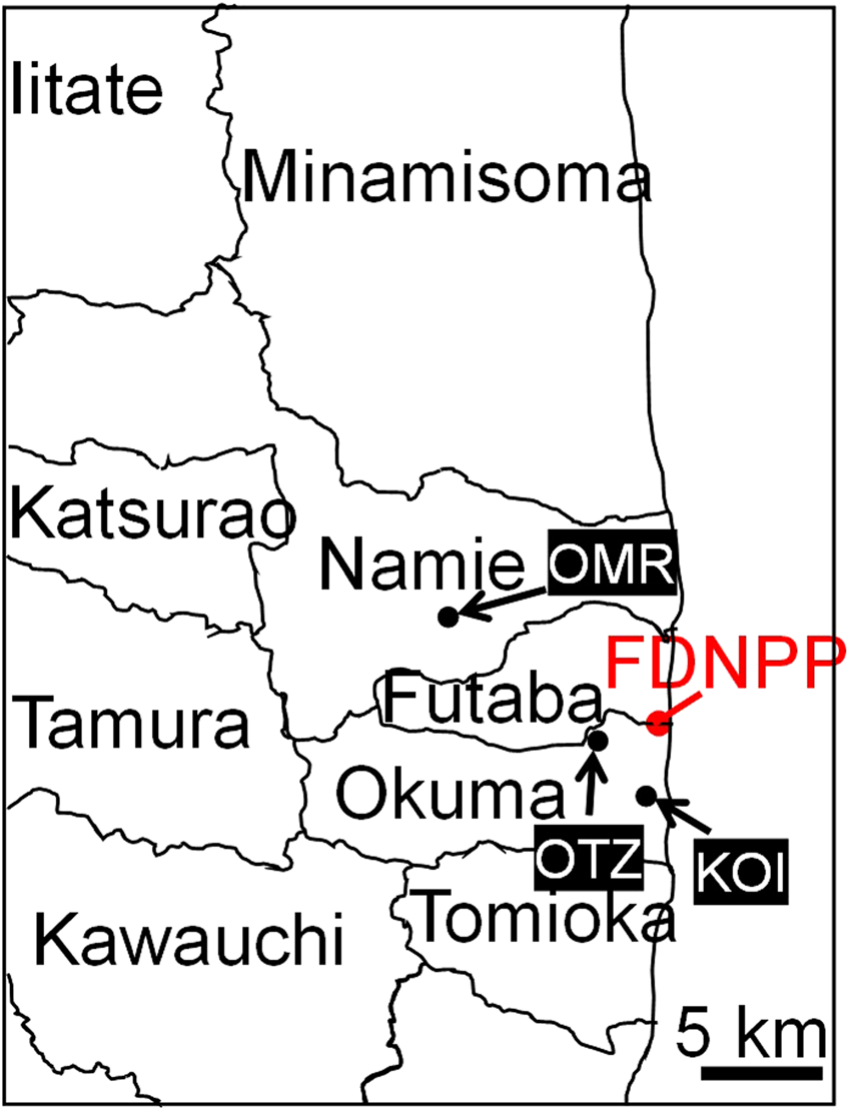
A map showing the location of the samples. This map was produced based on Furuki *et al*.^16^ using the power point.

**Figure 2:**
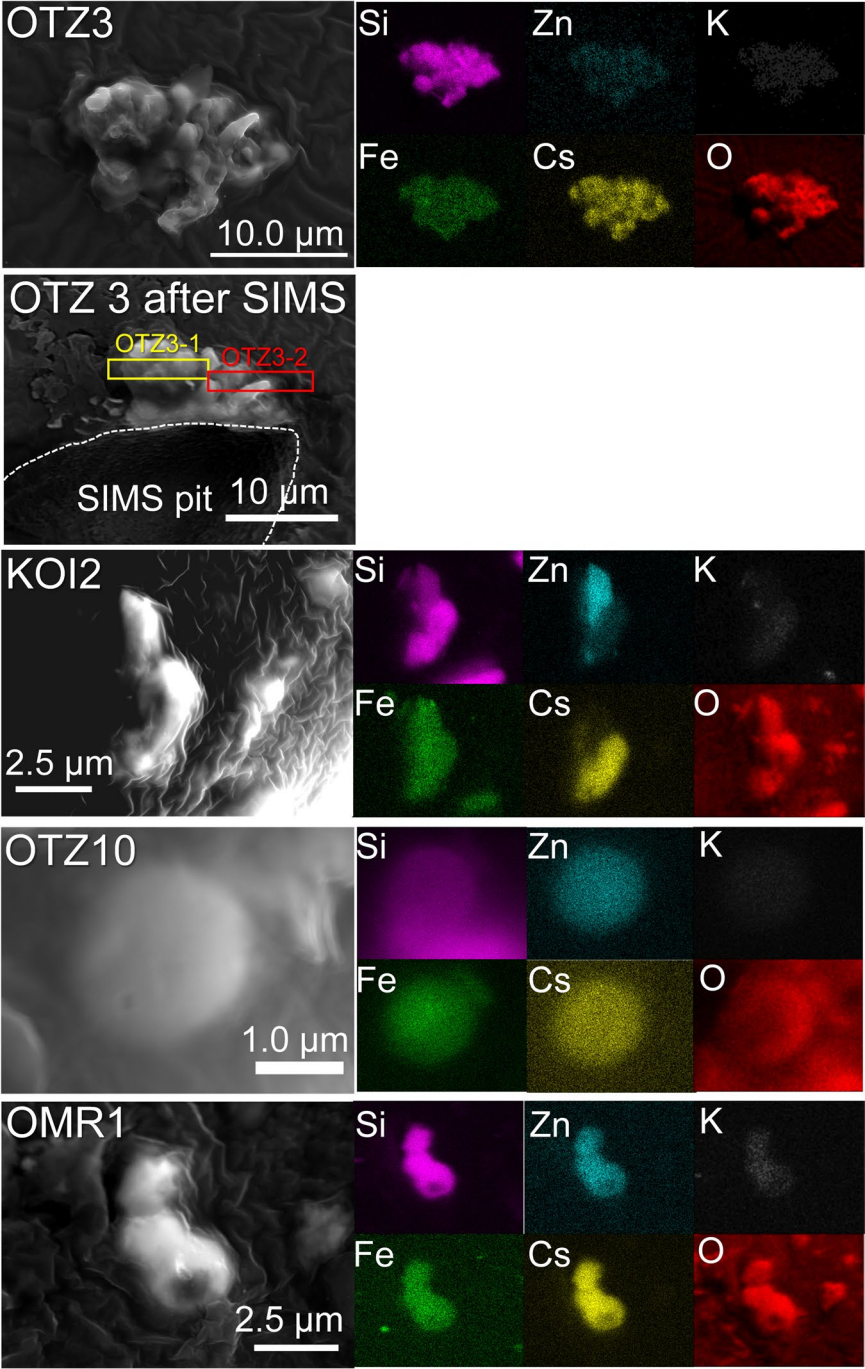
SEM images of CsMPs associated with the elemental maps.

Four CsMPs were found at three localities, which are hereafter labeled as OTZ3, OTZ10, KOI2, and OMR1 (Fig. 1). Detailed information on the sampling is provided in the method section. Figure 2 shows secondary electron scanning electron microscopy (SEM) images and energy-dispersive X-ray analysis (EDX) elemental maps of major elements of the CsMPs. In previous studies^12–16^, the shape of CsMPs has been described as spherical. In contrast, these particles appear as irregularly shaped aggregates rather than spheres. In addition, the radioactivity of the presently examined CsMPs ranges from 3.79 to 780 Bq (Table 1). These values are approximately thirty three times higher than that of spherical CsMPs^12–16^. The ^134^Cs/^137^Cs radioactivity ratios of the OTZ3, KOI2, and OMR1 used for the SIMS analysis are 1.06–1.08 (average 1.07), which approximately corresponds to ~26 GWd/tU according to OrigenArp calculations^18^. Although the ^134^Cs/^137^Cs isotopic ratio values are close to those of the reactor Units #2 and #3 in the FDNPP^19^, the specific reactor source could not be determined based only on the isotopic ratios, because burnups of the irradiated fuels in each reactor are heterogeneous depending on the positions of the fuel assemblies.

**Table 1:** Radioactivity of four CsMPs described in the present study.

Sample	Particle size (μm)	Radioactivity of ^134^Cs (Bq)	Radioactivity of ^137^Cs (Bq)	^Ratio of 134^Cs/^137^Cs ^radioactivity^
OTZ3	<17.3	401(±3.7)	379(±1.5)	1.06
OTZ10	2.2	1.85(±0.080)	1.94(±0.031)	0.955
OMR1	<5.2	21.5(±0.50)	20.3(±0.19)	1.06
KOI2	<4.4	18.2(±0.16)	16.8(±0.05)	1.08

The radioactivity was decay-corrected to March 12, 2011, 15:36 JST. The uncertainties in the radioactivity analyses are given in the parenthesis. The particle size was measured using the SEM image.

### Isotopic ratio based on secondary ion mass spectrometry (SIMS) and γ spectrometry

The results of the isotopic analysis of the three CsMPs, OTZ3, KOI2, and OMR1, are summarized in Table 2. In general, the ^235^U/^238^U isotopic ratio in nature is 0.00729, whereas the ratio in non-irradiated nuclear fuel is typically >~0.03 due to enrichment of ^235^U^20^. As shown in Table 2, U isotopes were successfully determined to be 0.029584 and 0.029341 in OTZ3 and KOI2, respectively, whereas U was not detected in OMR1. The U isotopic ratio for the standard specimen NIST SRM610 is comparable to the value in a previous report^21^, and the deviation of the three spot analyses with a standard deviation of *ca*. 0.00002 is 0.000001, indicating the high precision of the SIMS analysis.

**Table 2:** Summary of isotope analysis for three CsMPs using SIMS and γ spectrometry.

Isotopic ratio	OTZ3 (1σ)	KOI2 (1σ)	OMR1 (1σ)	Natural abundance ratio	SRM610 (1σ)
^235^U/^238^U	0.029584 (0.004967)	0.029341 (0.003032)	n/d	0.00729	0.002388 (0.000001)
^134^(Cs + Ba)/^137^(Cs + Ba)	0.02488 (0.00219)	0.03184 (0.00107)	0.03167 (0.00037)	—	—
^134^Cs/^137^Cs(γ)	0.0131	0.0135	0.0132		
^134^Ba_radiogenic_/^137^Ba_radiogenic_(γ)	0.515	0.517	0.520		
^134^Cs/^134^Ba_radiogenic_(γ)	0.190	0.190	0.191		
^137^Cs/^137^Ba_radiogenic_(γ)	7.47	7.46	7.47		
^134^(Cs + Ba_radiogenic_)/^137^(Cs + Ba_radiogenic_) (γ)	0.0724	0.0742	0.0727		
^135^Cs/^137^(Cs + Ba_radiogenic_)	0.3929 (0.0089)	0.4007 (0.0050)	0.4011 (0.0020)	—	—
^135^Cs/^133^Cs	0.3901 (0.0121)	0.3685 (0.0062)	0.3777 (0.0025)	—	—
^137^(Cs + Ba_radiogenic_)/^133^Cs	0.9941 (0.0543)	0.9199 (0.0246)	0.9417 (0.0102)	—	—
^134^(Cs + Ba_radiogenic_)/^138^Ba	36.6 (3.7)	35.3 (5.9)	137.7 (21.7)	0.03371	0.03371 (0.00009)
^136^Ba/^138^Ba	0.1523 (0.0124)	0.1638 (0.0191)	0.4293 (0.0697)	0.1095	0.1097 (0.0002)
^87^Rb/^85^Rb	2.407 (0.014)	2.284 (0.017)	2.236 (0.120)	0.3856	0.3857 (0.00009)
^90^Sr	n/d	n/d	n/d	—	—
^41^K/^39^K	0.07217 (0.00001)	—	—	0.07216	0.07214 (0.00003)
^40^K/^39^K	0.000127 (0.000041)	—	—	0.000125	—
^43^Ca/^44^Ca	0.06465 (0.00115)	—	—	0.06472	0.06469 (0.000005)

A NIST standard, SRM610, which contains depleted U, was also analyzed as a reference. Ten scans were conducted for each analytical spot on the CsMPs, meaning that the sequence of ten analyses represents a depth profile of the variation in the isotopic ratios; thus, SIMS analyses provide the isotope signatures inside the CsMPs. The average values are given in the table with the standard deviation calculated for the ten analyses and the standard deviations are given in the parenthesis as 1σ. The isotopic ratios recalculated from the results of γ spectrometry are annotated as (γ). Conversion of radioactivity to isotopic ratio was conducted by decay-correcting to the time of SIMS analysis. n/d stands for not detected.

Stable Cs is represented by monoisotopic ^133^Cs (100% natural abundance), although various radioisotopes including ^134^Cs, ^135^Cs and ^137^Cs are produced in a nuclear reactor, and the ratios of the isotopes depend on burnup. ^130^Ba (0.1058 at.%), ^132^Ba (0.1012 at.%), ^134^Ba (2.417 at.%), ^135^Ba (6.592 at.%), ^136^Ba (7.853 at.%), ^137^Ba (11.232 at.%), and ^138^Ba (71.699 at.%) are stable isotopes in nature (their abundance in at.%)^22^. Most Ba radioisotopes (>^122^Ba) typically decay through isobars to the most stable isotope in various modes such as β^−^, β^+^, and electron capture. Cesium and Ba isotopes are difficult to distinguish from one another in the present SIMS analysis. However, based on gamma spectrometry, the isotopic ratio of ^134^Cs/^137^Cs(γ) after decay-correction to the time of the SIMS analysis were calculated to be 0.0131, 0.0135, and 0.0132 for OTZ3, KOI2, and OMR1, respectively. The ^134^Cs/^137^Cs(γ) isotopic ratio after decay-correction to 15:36 March 12, 2011 was ~0.073. The ^136^Ba/^138^Ba isotopic ratio was determined to be 0.1523, 0.1638, and 0.4293 for OTZ3, KOI2, and OMR1, respectively. The isotopic ratio of ^134^(Cs + Ba_radiogenic_)/^138^Ba determined by SIMS ranges from 35 to 138. The isotopic ratios ^135^Cs/^137^(Cs + Ba_radiogenic_) and ^137^(Cs + Ba_radiogenic_)/^133^Cs were determined to be 0.39–0.40 and 0.92–0.99, respectively. The Rb isotopic ratio, ^87^Rb/^85^Rb, ranges 2.2–2.4. Strontium-90 is not detected in the CsMPs, and the isotopic ratios of Ca and K reveal that these elements are natural in origin.

### Nanoscale structure and composition of CsMPs

Two FIB thin sections were successfully prepared from the OTZ3 CsMP labeled OTZ3-1 and OTZ3-2, which were previously analyzed by SIMS. Another FIB specimen was prepared from OTZ10 CsMP that was found in the same soil sample.

**Figure 3:**
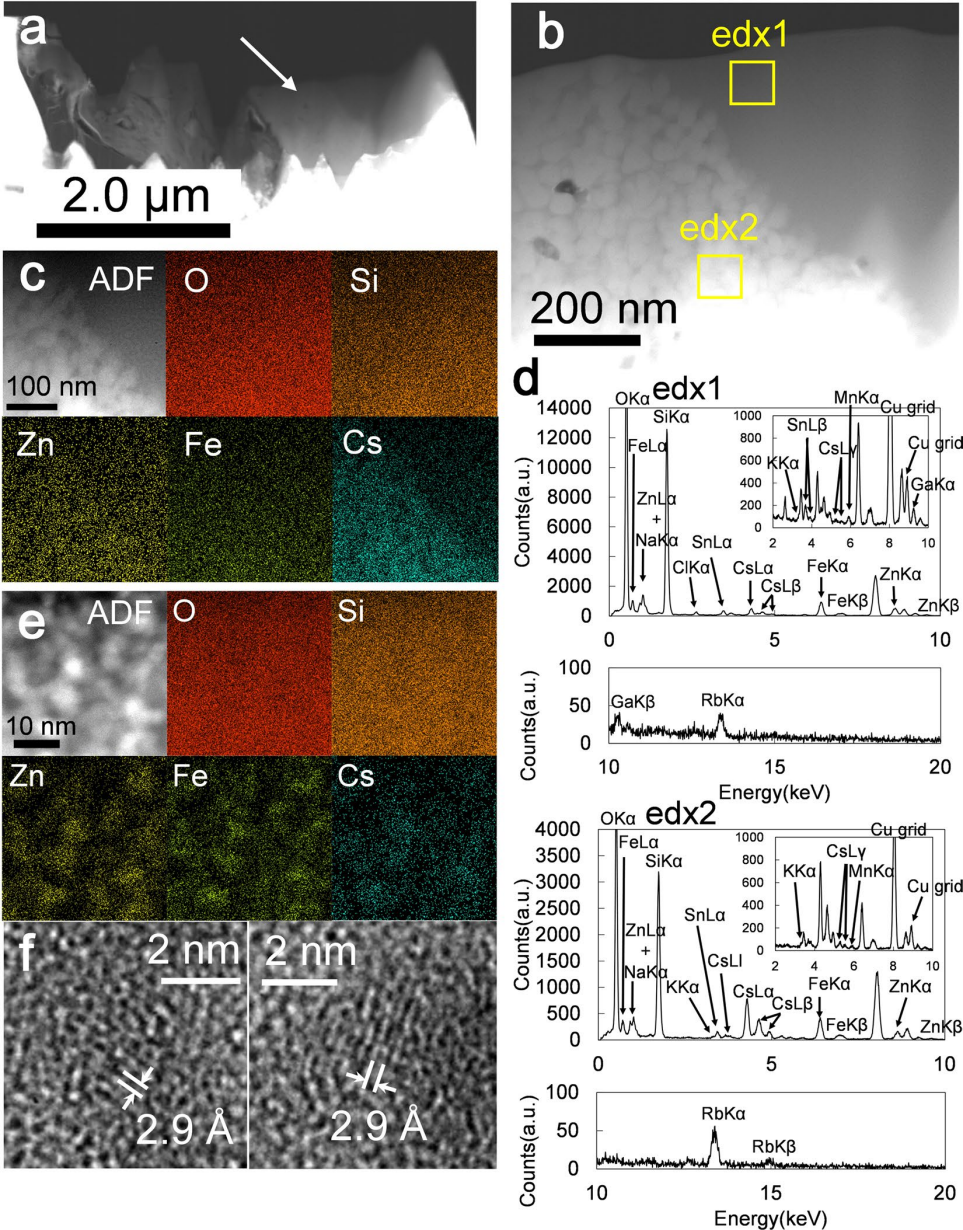
(**a**) A HAADF-STEM image of FIB-TEM specimen of OTZ3-1 CsMP. (**b**) Magnified HAADF-STEM image of the area indicated by the arrow in (**a**). (**c**) HAADF-STEM image (labeled as ADF) and the elemental map of the area displayed in (**b**). (**d**) STEM-EDX spectrums of the area indicated by the yellow square in (**b**). (**e**) Magnified HAADF-STEM image of the dark contrast zone represented as edx1 in (**b**) associated with elemental maps. (**f**) HRTEM image of the Zn–Fe-oxide nanoparticle. The lattice fringe of 2.9 Å corresponds to (220).

A high-angle annular dark-field scanning transmission electron microscopy (HAADF-STEM) image of OTZ3-1 CsMP is shown in Fig. 3a, in which the white arrow indicates the position of the particle. There are two distinct zones with different contrast (Fig. 3b). The elemental maps of the boundary between the two zones reveal that the Cs concentration is much higher in the area with bright contrast, although the other elements are distributed homogeneously (Fig. 3c, Table S1). The area with dark contrast (edx1) contains a lower Cs-content as compared with the zone with bright contrast (edx2) (Fig. 3d). The magnified HAADF-STEM image of the low Cs zone (dark contrast) is composed of nanoparticles that consist of Zn and Fe, as revealed by the elemental maps (Fig. 3e). Cesium is associated with these nanoparticles. The distribution of silicon could not be resolved due to the thickness of the specimen (Fig. 3e). Based on the HRTEM image, the nanoparticles are most likely identified as franklinite structures (ZnFe_2_O_4_, *Fd*3*m*, *Z* = 8)^23^, which has also been identified in other CsMPs^15, 16^ (Fig. 3f).

The high Cs zone (represented by edx2 in Fig. 3b) appears to contain relatively large nanoparticles, ~50 nm in size, with a high Cs-content (Fig. S1a) as compared with the franklinite nanoparticles found in the low Cs-zone. The large Cs-rich particle was subsequently identified as an Fe-rich pollucite structure (CsFeSi_2_O_6_ · nH_2_O, *Ia*3*d*, *Z* = 16)^24^ based on the SAED pattern (Fig. S1b). Pollucite is a zeolite with the ideal formula (Cs,Na)(Al,Si)_3_O_6_ · nH_2_O^25^. The Fe-pollucite is unstable under electron beam irradiation and becomes amorphous (Fig. S1c), possibly due to the loss of water. In addition, the HAADF-STEM image of the boundary between the high- and low-Cs zones displays different levels of contrast after electron beam irradiation (Fig. S1d); that is, the bright contrast of Fe-pollucite particles disappears, indicating the structural degradation of Fe-pollucite, consistent with the loss of peaks in the SAED pattern. Electron energy-loss spectroscopy (EELS) of the high-Cs zone reveals that Cs is monovalent as indicated by the Cs M-edge (Fig. S1e). Barium is closely associated with Cs-bearing phases rather than forming a separate Ba-rich phase, which is expected since almost all Ba isotopes in the CsMP are decay daughters of radioactive Cs. The iron *L*-edge confirms that Fe is present in the form of oxides.

**Figure 4:**
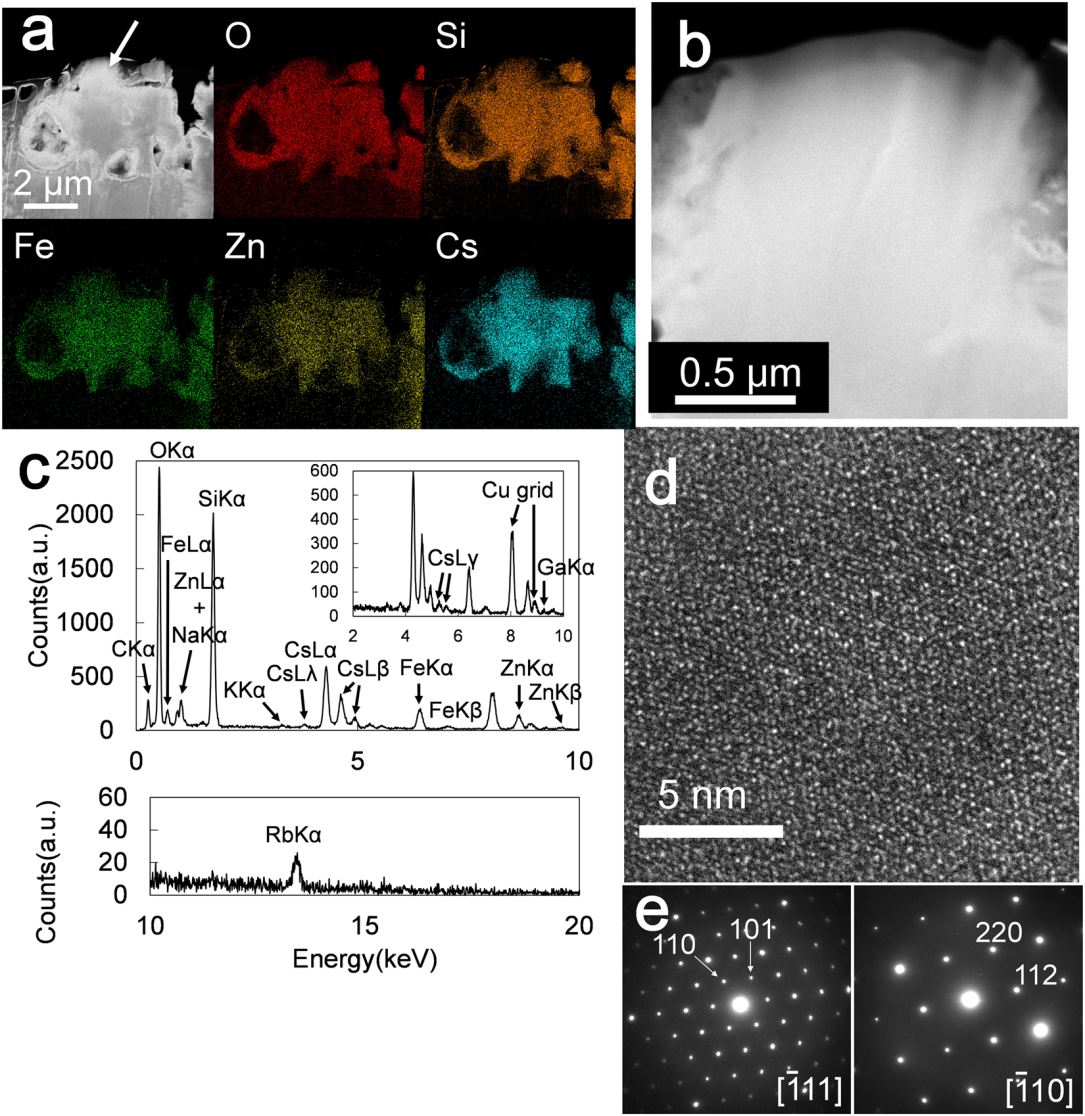
(**a**) HAADF-STEM image of the FIB-TEM specimen OTZ3-2 associated with the elemental maps. (**b**) A magnified HAADF-STEM image of the high Cs phase indicated by the arrow in (**a**). (**c**) A representative EDX spectrum of the high Cs phase indicated by the arrow in (**a**). (**d**) HRTEM image of the high Cs phase indicated by the arrow in (**a**). (**e**) The SAED pattern of the high Cs phase from two different major zone axes.

The same major elements, Si, Fe, Zn, and Cs, are present in the other FIB specimen from the same aggregate, OTZ3-2, as well (Fig. 4a). The area indicated by the white arrow is magnified, and the texture is rather homogenous, as shown by the HAADF image contrast (Fig. 4b), which is different from the spherical texture observed in the high-Cs zone (edx2 in Fig. 3b) in OTZ3-1. However, the Cs-content (Fig. 4c) is similar to that of the high-Cs zone in OTZ3-1. Also observed was a single crystal as large as ~0.5 μm, evidenced by continuous lattice fringes in the HRTEM image (Fig. 4d). Based on the diffraction patterns obtained from the two major zone axes, this large Cs-phase is an Fe-pollucite (Fig. 4e).

**Figure 5:**
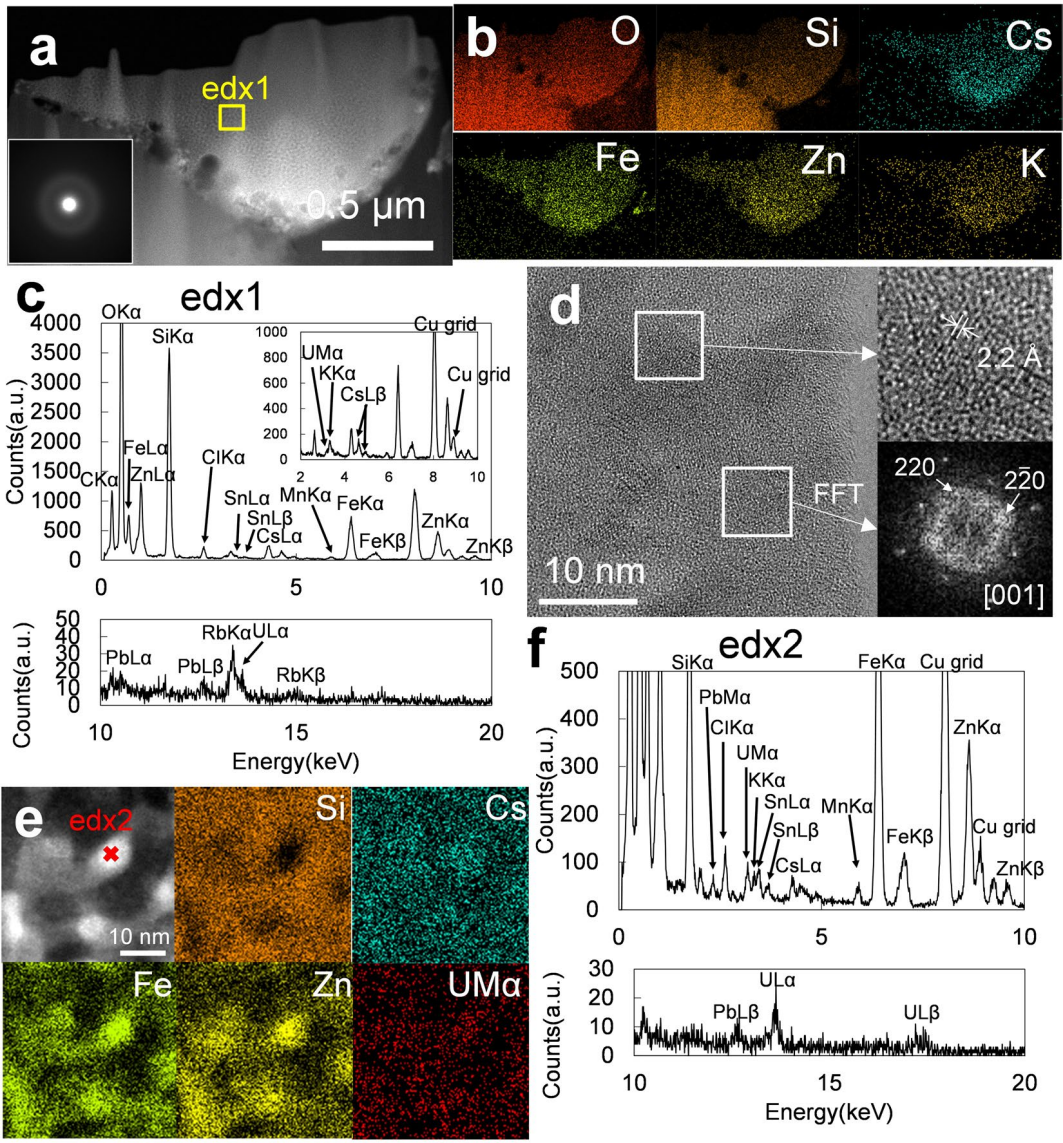
(**a**) HAADF-STEM image of the FIB-TEM specimen of the OTZ10 CsMP with the SAED as inset. (**b**) Elemental maps of the same area as the HAADF-STEM image in (**a**). (**c**) The EDX spectrum of the area indicated by the yellow square labeled as edx1 in (**a**). (**d**) A HRTEM image of the Zn–Fe-oxides nanoparticles accompanied with the magnified lattice image and the FFT image of the selected area indicated by the white square. The lattice fringe of 2.2 Å corresponds to (400). (**e**) HAADF-STEM image of the enlarged figure of the thin edge in (**a**) associated with elemental maps. (**f**) STEM-EDX point analysis on a Zn–Fe-oxide nanoparticle indicated by a red cross labeled as edx2 in (**e**).

In order to determine the presence of U, a FIB-prepared cross-section of the OTZ10 CsMP was additionally investigated by TEM (Fig. 5a). Typically, the SAED exhibits broad diffraction maxima that correspond to diffuse scattering from amorphous domains (Fig. 5a inset). The elemental map of the entire cross-section displays a homogeneous distribution of the major constituents at the bulk scale (Fig. 5b). The Cs-concentration is 7–10 wt% (Fig. 5c, Table S1). Despite the apparently homogeneous distribution and the diffuse scattering halo in the SAED, the magnified HAADF-STEM image shows phase separation as evidenced by differences in contrast (Fig. S2a). The elemental map and the EDX analysis indicate that the phase in bright contrast contains Fe, Zn, Cs, and Sn; whereas, the area of dark contrast corresponds to SiO_2_ -domains (Fig. S2b and c). The HRTEM image also reveals the presence of many nanocrystals (Fig. 5d). Based on the d-spacing and the FFT image, these nanocrystals are franklinite^23^. Another magnified HAADF-STEM image with an elemental map reveals that the U distribution is closely associated with Zn–Fe-oxide nanoparticles (Fig. 5e). The U concentration is 0.8 wt% (including Si) or 1.1 wt% (excluding Si), assuming U is in the form of U_3_O_8_ for the quantitative EDX analysis (edx2, Fig. 5f).

## Discussion

The U isotopic ratios of the ten scans on the CsMPs vary with a rather large deviation, ~0.0050 for OTZ3 and ~0.0030 for KOI2, most likely because the U concentration in the CsMPs is low (ppm level in the bulk). In addition, the analyses performed are not sufficiently precise to provide the absolute concentrations as high as that of the analysis on SRM610, because the irregular surfaces of the CsMPs aggregate were analyzed without polishing. The effect of isotopic fractionation should be negligibly small during the formation of these CsMPs. Even with a large deviation in the isotopic ratios, ^235^U/^238^U in OTZ3 and KOI2 are significantly high, 0.029584 and 0.029341, as compared with the U isotopic ratio in nature, 0.00729. As expected, the U isotopic ratios are close to that of a typical nuclear fuel enriched in ^235^U.

The presence of ^138^Ba indicates either a fissionogenic or natural origin. Natural ^136^Ba/^138^Ba, 0.1095, is an order of magnitude greater than ^136^Ba/^138^Ba of fissionogenic origin, ~0.0126, estimated for Units #1–3 by calculation using the ORIGEN code^19^, which largely varies depending on the burnup: 0.004 for 10 MWd/kgU, 0.011 for 20 MWd/kgU, and 0.014 for 30 MWd/kgU. ^136^Ba/^138^Ba is 0.1523, 0.1638, and 0.4293 for OTZ3, KOI2, and OMR1, respectively. These values are much higher than those of fissionogenic Ba. The ^136^Ba/^138^Ba ratio may indicate the contribution of a low-yield fission product; the amount of ^136^Cs (half-life of 13.16 days) can be remarkable in some cases depending on the amount of volatilized Ba. Although the amount of fissionogenic Ba isotopes is greater than Cs isotopes, the Cs can volatilize to greater extent than Ba during the meltdown, meaning that CsMP can selectively incorporate Cs as compared with Ba. Because ^136^Ba is a decay product of ^136^Cs (half-life of ~13 days), ^136^Ba was the most likely incorporated in the form of Cs species, leading to the higher value of ^136^Ba/^138^Ba. On the other hand, ^134^(Cs + Ba)/^138^Ba determined by SIMS varies from 35 to 138, which is much higher than ^134^Ba/^138^Ba in nature (0.03371) and that of fissionogenic origin (0.0373–0.0566). Based on results from calculations using the ORIGEN code^19^, the initial amount of fissionogenic ^134^Cs should be about the same as ^134^Ba. Thus, the high ^134^(Cs + Ba)/^138^Ba is probably due to the higher volatility of Cs^26^, as well as the greater amount of Cs incorporated into the CsMPs as compared with Ba. Thus, Ba isotopes of natural and fissionogenic origin that were initially incorporated in the CsMPs are negligibly small. Rather, ^134^Ba and ^137^Ba are decay daughters derived from Cs isotopes. ^134^Cs/^137^Cs (γ) isotopic ratio at 15:36 March 12, 2011 was ~0.073, and based on the calculation of decayed amount of ^134^Cs and ^137^Cs corresponding to that of ^134^Ba and ^137^Ba in the CsMPs, the two isotopic ratios, ^134^Cs/^134^Ba_radiogenic_(γ) and ^137^Cs/^137^Ba_radiogenic_(γ) at the time of the SIMS analysis, were ~0.19 and ~7.47, respectively (Table 2).

Since the amount of volatilized Ba is negligible and the Ba in the CsMPs is mostly radiogenic, ^135^Cs/^133^Cs determined by SIMS represents the initial isotopic ratio, because ^133^Cs is a stable isotope and ^135^Cs has a long half-life, 2.3 million years. The previous ORIGEN calculation reported ^135^Cs/^133^Cs to be 0.388, 0.344, and 0.353 for Units # 1, 2, and 3, respectively, which are close to the present SIMS results, 0.39, 0.37, and 0.38 for OTZ3, KOI2, and OMR1, respectively. The isotopic ratios ^135^Cs/^137^(Cs + Ba_radiogenic_) and ^137^(Cs + Ba_radiogenic_)/^133^Cs calculated by SIMS are 0.39–0.40 and 0.92–0.99, respectively, which are comparable to the initial ^135^Cs/^137^Cs and ^137^Cs/^133^Cs values calculated by ORIGEN, 0.40 and 0.98, 0.34 and 1.01, 0.35 and 1.01, for Units #1, 2, and 3, respectively. Moreover, ^135^Cs/^137^(Cs + Ba_radiogenic_) is also comparable to ^135^Cs/^137^Cs of the bulk soil samples previously analyzed using thermal ionization mass spectrometry (TIMS), 0.36–0.38^27, 28^. Because of the high ^137^Cs/^137^Ba_radiogenic_, the majority of 137-mass derives from ^137^Cs, and the efficiency of secondary ions formation for ^137^Ba should be minimal.

The values of ^134^(Cs + Ba_radiogenic_)/^137^(Cs + Ba_radiogenic_) (γ) determined through gamma spectrometry (0.0724–0.0742), which are the same as those of the initial ^134^Cs/^137^Cs, are higher than the values of ^134^(Cs + Ba)/^137^(Cs + Ba) for CsMPs that were determined by SIMS (0.0249–0.0318). This is attributed to the difference in ionization efficiency between Cs and Ba. Introducing the secondary ion production efficiency coefficient, ^i^ *f* = ^i^Ba ionization efficiency/^i^Cs ionization efficiency, where i stands for the mass, the ratio by SIMS can be expressed as

1 $$\begin{array}{c}({}^{{\rm{134}}}{\rm{C}}{\rm{s}}+{}^{134}{\rm{B}}{{\rm{a}}}_{{\rm{radiogenic}}}\times {}^{134}f)/({}^{137}{\rm{C}}{\rm{s}}+{}^{137}{\rm{B}}{{\rm{a}}}_{{\rm{radiogenic}}}\times {}^{137}f)=\,0.029\,({\rm{average}}\,{\rm{for}}\,{\rm{three}}\,{\rm{CsMPs}})\end{array}$$

Because the difference between ^134^ *f* and ^137^ *f* is negligible, the ratio of the ionization efficiency is simply expressed as *f*, and equation (1) can be rewritten as

2 $$\begin{array}{c}({}^{{\rm{134}}}{\rm{C}}{\rm{s}}+{}^{{\rm{134}}}{\rm{B}}{{\rm{a}}}_{{\rm{radiogenic}}}\times f)/({}^{137}{\rm{C}}{\rm{s}}+{}^{137}{\rm{B}}{{\rm{a}}}_{{\rm{radiogenic}}}\times f)\\ \quad =\,{({}^{134}{\rm{C}}{\rm{s}}/{}^{137}{\rm{C}}{\rm{s}})}_{{\rm{initial}}}(0.16+0.84\times f)/(0.88+0.12\times f)\\ \quad =\,0.029\end{array}$$

Solving equation (2): *f* = 0.24.

This ionization efficiency factor is reasonably close to that determined for rhyolitic glass (SRM610); 0.288. Note, the ionization efficiency of the standard specimen is determined on the polished flat surface on the pure glass, while that for the CsMP was determined on the spherical shaped particle that is a mixture of SiO_2_ glass and the Zn–Fe-oxides nanoparticles. Thus, the matrix composition is similar but not identical.

The Rb isotopic ratio, ^87^Rb/^85^Rb, is 2.2–2.4, which is obviously higher than the natural ^87^Rb/^85^Rb (0.3856), indicating that the Rb isotopes are fissionogenic. Compared with the ^87^Rb/^85^Rb values calculated by ORIGEN^19^ (~1.7), ^87^Rb/^85^Rb obtained by SIMS is relatively high. Nevertheless, our OrigenArp calculation gives ratio values between 2.5 and 2.7, which are closer to the ^87^Rb/^85^Rb values by SIMS. The difference is not ascribed to the mixture of natural Rb isotopes because the mixing with the natural isotopes would lower the ^87^Rb/^85^Rb isotopic ratio, but rather due to the local volatilization of Rb.

The nanoscale texture of OTZ10 and the low-Cs zone in OTZ3-1 reveal that franklinite nanoparticles are embedded in a glassy matrix of SiO_2_. These textures and the Cs association with franklinite were also reported in previous studies^15, 16^, indicating multiple steps in the formation sequence: radioactive Cs is released to form nanoparticles and/or is present as droplets in a mist during meltdown; subsequently, numerous Zn–Fe-oxide (franklinite) nanoparticles formed during the failure of the reactor pressure vessel (RPV), and Cs in wet form is adsorbed onto the Zn–Fe-oxide nanoparticles; then, the melted fuels break through the RPV, hit the concrete pedestal, and generate SiO gas at >2,000 K. This process is known as the molten core-concrete interaction (MCCI)^29^, which immediately leads to the condensation of SiO_2_ over the Zn–Fe-oxide nanoparticles and the incorporation of fission product nanoparticles. As shown in a previous study^16^, a low concentration of U (~1 wt%) is observed associated with franklinite nanoparticles that formed before the CsMP formation. Moreover, U adsorption onto the franklinite nanoparticles possibly occurs through volatilization of the oxidized form of the UO_2_ fuel. In fact, UO_2_ pellets can be oxidized depending on the H_2_O:H_2_ ratio^30^. In general, U is considered a nonvolatile element; however, the partially oxidized form of UO_2+X_ can volatilize by ~10% at >~1,900 K at a heating rate of ~30 K/min; whereas, the unoxidized form UO_2_ does not become volatile even at 2,700 K^31, 32^. Uranium association with microscale aerosols during a severe accident was also recognized in some experimental studies^33^. The chemical form of U inside CsMPs has been determined in the OTZ10 CsMP, as well as in the other CsMP separated from the same soil sample^16^. The U isotopic ratio in another CsMP, OTZ3, found in the same soil sample, was determined. ^235^U/^238^U = ~0.030, as analyzed from the two CsMPs, indicating that the U incorporated in the CsMPs is not from naturally occurring trace U that has a ^235^U:^238^U ratio of 0.00729:1, which was initially considered as the source of U because trace amounts of U are included in the insulating materials surrounding reactor pressure vessel. Rather, nuclear fuel is the most likely origin of U in the CsMPs. The original isotope composition of non-irradiated fuel before operation at the FDNPP was 0.0389^19^. Based on calculations using the ORIGEN code, the U isotopic ratios for the irradiated fuel in Units # 1, 2 and 3 were 0.0172, 0.0193, and 0.0192^19^, respectively. Thus, the isotopic ratios are between the isotopic ratio calculated from burnup and that of the non-irradiated fuel. The isotopic ratios calculated by the ORIGEN code are based on the average burnup for all fuel assemblies. The intermediate values of ^235^U/^238^U are due to the fact that the burnup and the temperature are not homogeneous within the reactor core. A fuel assembly will experience different temperatures depending on its position in the core. Even within a single pellet, there is a thermal gradient; the center of the pellet can be heated as high as ~1,973 K, whereas the temperature of the rim is only ~673 K^34^. Moreover, the burnup within a single pellet is not uniform with low burnup at the center and high burnup at the edge^34^. The burnup of the fuel assembly is not uniform either, as the fuel rods with different burnup are assembled in a certain matter. Thus, the fuel rods with relatively lower burnup might have been heated at the temperature at which a small amount of U was volatilized, resulting in the high ^235^U/^238^U isotopic ratios for both CsMPs. The volatilized U is associated with Zn–Fe-oxide, most likely by simple adsorption, as evidenced by the U in the OTZ10 CsMP.

Dispersion of U released from the FDNPP has been reported in previous studies^35–37^, including ^236^U/^238^U values of ~10^−9^ in paddy-field water and ocean water as far as ~30 km from the FDNPP^35^. ^236^U/^238^U values of ~10^−7^ were also reported in black-colored dust samples collected within a ~30-km distance^36^. This is evidence that a small amount of U, about 150 g^36^, was discharged from the fuels in the reactors. Nevertheless, another study measuring ^235^U/^238^U failed to identify the FDNPP as the origin due to the dilution by natural U isotopes^37^. However, all those analyses were performed on bulk soil samples and did not include data from single CsMPs. Thus, the speciation of U was never determined. The present study convincingly identifies the U speciation as associated with Zn–Fe-oxide nanoparticles at ~1 wt%, and the nuclear fuel origin of U in CsMPs is confirmed through isotopic ratio analyses. Yet, quantitative analysis of the amount of U in the form of CsMPs in the total U that was dispersed to environment remains undetermined.

Two different occurrences of radioactive Cs have been identified: Fe-pollucite and Cs associated with franklinite nanoparticles embedded in the glassy SiO_2_ matrix. The latter has been already described above and in previous studies^15, 16^ and is not discussed further here. On the other hand, Fe-pollucite, CsFeSi_2_O_6_ · nH_2_O, is identified for the first time in CsMPs. None of the previous studies reported pollucite during meltdown; however, a recent experimental study reported formation of pollucite and CsFeSiO_4_ during the CsOH chemisorption onto stainless steel containing 5% Si^38^. Both materials have a zeolite structure that can be synthesized by annealing a mixture of gels^39^ and appropriate elements, such as Cs^40^. Their formation by chemisorption likely occurred at the high-Cs zone during the formation of CsMPs. However, the reaction required for CsMPs formation are apparently different from the simple interaction of CsOH with stainless steel at ~1,273 K^38^. In fact, the Cs-Cr phases that result from the reaction with stainless steel are not present in CsMPs. Rather, it is probable that CsOH is involved in the chemical reactions with Si and Fe oxide during the MCCI at >2,000 K. The clear boundary between the high- and low-Cs zones (Fig. 3b) indicates that Zn–Fe-oxide nanoparticles formed aggregates prior to the formation of Fe-pollucite, which formed at the time of MCCI.

In summary, the present study successfully determined the origin of U, Cs, Ba, Rb, K, and Ca in the CsMPs, as well as their chemical form based on the isotopic ratios and their nanoscale structure. In particular, the data obtained are critically important for delineating the reactions involving nuclear fuels, which occurred inside the reactor during the formation of the CsMPs. The detailed information on the chemical state and source provide an understanding of the source term at the FDNPP during the meltdown event. This is important information for severe accident (SA) analysis codes, such as MELCOR^41, 42^ and MAAP^43^, since some of the phases identified in the CsMPs, such as Zn–Fe-oxide and pollucite, are not considered in these severe accident codes^44^.

## Methods

### Sampling

We collected the samples at three locations (Fig. 1). As for the sample labels, the capital letters represent the locality and the following number indicate the number in our CsMP database in Kyushu University. The OTZ3 and OTZ10 were separated from the same soil collected from the top ~1 cm of the paddy soil at Ottozawa located ~4 km west from the FDNPP in Okuma Town, Futaba County, Fukushima, on March 16, 2012. The soil was mainly composed of clay minerals, quartz, and feldspar^9^. Because it is still prohibited to enter the area due to the high radiation dose, the locality remained untouched and was not disturbed by decontamination or restoration. The radiation dose ~1 m above the ground was measured at 84 μSv/h.

The second sample (KOI2) was composed of gravels collected under the drainpipe of the assembly house in Koirino located 2.9 km southwest from the FDNPP during the same sampling campaign. The radiation dose underneath the drainpipe was extremely high compared to the surroundings; the dose at the sampling point was as high as 630 μSv/h. The gravel samples were carefully collected from the ground surface using a hand shovel and placed in plastic bags. The soil was also mainly composed of clay minerals, quartz, and feldspar^9^. The third sample (OMR1) was collected under the drainpipe of the warehouse in Omaru located in Namie Town, Futaba County, Fukushima, ~10.5 km northwest from the FDNPP during the sampling campaign on December 20, 2012. The radiation dose ~1 m above the ground exceeded 30 μSv/h.

### Separation of CsMPs

Prior to the procedure, both samples were sieved through a 114-μm mesh. The powder samples were dispersed on grid paper and then covered with a plastic sheet. Next, an imaging plate (IP, Fuji film, BAS-SR 2025) was placed on the samples for 5–15 min. Further, using an IP reader, autoradiograph images with pixel sizes of 100 μm were recorded. After identifying the positions of intense radioactive spots, droplets of pure water were added to these positions and then drawn using a pipette to produce suspensions with small amounts of soil particles by dilution with pure water. This procedure was repeated until the suspension did not contain a significant amount of soil particles. Subsequently, the positions containing hot spots were selected using pieces of double-stick carbon tape that were cut as small as possible with a blade. The pieces were checked by autoradiograph imaging to obtain CsMPs with maximum efficiency using scanning electron microscopy (SEM) observations. Prior to SEM analysis, the pieces were placed on an aluminum plate and coated with carbon using a carbon coater (SANYU SC-701C). The CsMPs were found and observed using two SEMs (Shimadzu, SS550 and Hitachi, SU6600) using acceleration voltages of 15–25 kV both equipped with an energy dispersive X-ray spectrometer (EDX, EDAX Genesis).

### Preparation of TEM specimens

A focused ion beam (FIB) instrument (FEI, Quanta 3D FEG 200i Dual Beam) was utilized to prepare a thin foil of individual CsMPs with diameters of a few μm. Gallium was used as an ion source, and W deposition was used to minimize the damage from the ion bombardment. The current and accelerating voltage of the ion beam were adjusted from 100 pA to 30 nA and 5–30 kV, respectively, depending on the progress of the thinning and sample properties such as hardness and size. Each thinned piece was attached to the semilunar-shaped Cu grid for FIB and further thinned by an ion beam operating at 5 kV.

### TEM analysis

HRTEM with EDX and high-angle annular dark-field scanning transmission electron microscopy were performed using a JEOL JEM-ARM200F and JEM-ARM200CF with an acceleration voltage of 200 kV. The JEOL Analysis Station software was used to control the STEM-EDX mapping. To minimize the effects of sample drift, a drift-correction mode was used during acquisition of the elemental map. The STEM probe size was ~0.13 nm, generating an ~140-pA current when 40 μm of the condenser lens aperture was inserted. The collection angle of the HAADF detector was ~97–256 mrad. A Gatan imaging filter system was used to conduct electron energy-loss spectroscopy in STEM mode. The convergent and collection semi-angles were ~30 mrad and ~17 mrad, respectively.

### Gamma spectrometry

The ^134^Cs and ^137^Cs radioactivities of the CsMPs were determined using gamma spectrometry. The radioactivity of an additional microparticle with a size of ~400 μm obtained from surface soil in Fukushima was precisely determined at the radioisotope center in Tsukuba University, Japan, and utilized as a standard point specimen for ^134^Cs and ^137^Cs. The radioactivity of the point source standard was 23.9 Bq for ^134^Cs and 94.6 Bq for ^137^Cs as of September 29, 2015. Measurement of the radioactivity was performed on the CsMPs and the point source standard using a germanium semi-conductor detector, GMX23 (SEIKO E&G), GMX40 (SEIKO E&G) and GX6020 (Canberra) at the center for radioisotopes in Kyushu University, Japan. The acquisition times were 21,238 s for OMR1 and 84,289 s for OTZ10 using GMX23, 4,004 s for OTZ3 using GMX40, and 77, 880.8 s for KOI2 using GX6020.

### Secondary ion mass spectrometry

Isotopic ratio analysis was performed using secondary ion mass spectrometry (SIMS, SHRIMP-II, Australian Scientific Instruments) at the National Institute of Polar Research, Tokyo. The SIMS specimens were put on Al plates or a Cu grid and fixed on 1-inch slide glass by Cu tape. They were coated with Au at a 13.5-nm thickness prior to the analysis. An O_2_^−^ primary ion beam of 0.2–0.4 nA was used to sputter the specimen surface with a beam diameter of 5.0–7.0 μm. The typical mass resolution is about 4,500 (M/ΔM at 1% of peak height). The National Institute of Standards and Technology (NIST) SRM610 was used as a standard specimen, in which 461.5 mg/kg of depleted U was doped in a silicate glass matrix.

Because quantitative analysis using SIMS requires suitable standards, which have chemical compositions similar to those of the target minerals, the absolute concentrations were not obtained, but only the isotopic ratios were determined. Ten scans were completed for each analytical spot on the CsMPs, meaning that the sequence of ten analyses represents a depth profile of the variation in the isotopic ratios; thus, SIMS analyses provide the isotopic signatures inside the CsMPs. The average values are given in the Table 2 with the standard deviation calculated for the ten analyses.

## Electronic supplementary material

Supplementary Information
