# Supplementary material for: Isotopic signature and nano-texture of cesium-rich micro-particles: Release of uranium and fission products from the Fukushima Daiichi Nuclear Power Plant
Source: Sci Rep. 2017 Jul 14;7:5409. doi: 10.1038/s41598-017-05910-z (PMC5511200; doi:10.1038/s41598-017-05910-z)
Supplement: Supplementary file 1 — Supplementary Information [file 41598_2017_5910_MOESM1_ESM.pdf]

Supplementary Information for

# Isotopic signature and nano-texture of cesium-rich micro-particles: Release of uranium and fission products from the Fukushima Daiichi Nuclear Power Plant

**Junpei Imoto [1,**], Asumi Ochiai [1,**], Genki Furuki [1], Mizuki Suetake [1], Ryohei Ikehara [1], Kenji Horie [2,3], Mami Takehara [2], Shinya Yamasaki [4], Kenji Nanba [5], Toshihiko Ohnuki [6], Gareth T. W. Law[7], Bernd Grambow [8], Rodney C. Ewing [9],**

**and Satoshi Utsunomiya [1*]**

In *Scientific Reports* **7**, DOI:10.1038/s41598-017-05910-z

Table S1. Chemical composition of the OTZ3-1 and OTZ10 CsMPs determined by STEM-EDX area analysis.

| | $SiO_2$ | $Cs_2O$ | $Fe_2O_3$ | ZnO | $Rb_2O$ | $K_2O$ | $SnO_2$ | PbO | MnO | Cl |
|---|---|---|---|---|---|---|---|---|---|---|
| OTZ3-1 | | | | | | | | | | |
| *High Cs zone* | | | | | | | | | | |
| Area1 | 48.0 | 29.6 | 9.15 | 3.56 | 3.79 | 0.37 | 5.10 | 0.02 | 0.32 | 0.07 |
| Area2 | 51.1 | 28.9 | 9.68 | 3.64 | 0.68 | 0.31 | 5.26 | 0.03 | 0.30 | 0.08 |
| Area3 | 58.0 | 19.2 | 10.4 | 4.25 | 1.71 | 0.25 | 5.34 | 0.02 | 0.59 | 0.21 |
| Area4 | 59.5 | 16.8 | 10.3 | 4.36 | 2.16 | 0.28 | 5.92 | 0.02 | 0.43 | 0.27 |
| Area5 | 62.6 | 13.7 | 10.3 | 4.03 | 1.76 | 0.14 | 6.87 | 0.01 | 0.53 | 0.07 |
| Area6 | 59.6 | 29.9 | 8.16 | 0.02 | 0.85 | 0.20 | 0.19 | 1.01 | 0.04 | 0.02 |
| Area7 | 62.6 | 15.3 | 11.3 | 7.01 | 1.66 | 0.16 | 1.19 | 0.50 | 0.18 | 0.06 |
| *Low Cs zone* | | | | | | | | | | |
| Area8 | 69.5 | 7.09 | 9.40 | 5.14 | 1.23 | 0.26 | 6.02 | 0.36 | 0.44 | 0.59 |
| Area9 | 72.7 | 6.01 | 7.85 | 4.86 | 1.04 | 0.34 | 5.67 | 0.40 | 0.48 | 0.64 |
| Area10 | 77.3 | 4.80 | 6.97 | 5.66 | 0.74 | 0.19 | 3.03 | 0.38 | 0.37 | 0.55 |
| Area11 | 74.2 | 5.42 | 8.05 | 4.65 | 0.99 | 0.24 | 5.02 | 0.26 | 0.53 | 0.62 |
| OTZ10 | | | | | | | | | | |
| Area1 | 57.1 | 8.18 | 16.2 | 11.7 | 1.82 | 1.09 | 1.74 | 0.02 | 0.76 | 1.42 |
| Area2 | 55.4 | 7.79 | 16.5 | 11.9 | 2.01 | 1.50 | 2.37 | 0.26 | 0.81 | 1.52 |
| Area3 | 54.3 | 7.16 | 18.2 | 13.2 | 1.61 | 1.71 | 1.44 | 0.34 | 0.50 | 1.55 |
| Area4 | 55.7 | 9.92 | 15.5 | 11.7 | 1.42 | 1.37 | 1.68 | 0.63 | 0.69 | 1.43 |
| Area5 | 54.1 | 8.16 | 17.0 | 13.0 | 0.90 | 1.57 | 1.69 | 1.21 | 0.86 | 1.51 |
| Area6 | 59.9 | 7.64 | 16.4 | 10.9 | 0.43 | 0.84 | 1.88 | 0.01 | 0.69 | 1.30 |
| Area7 | 61.6 | 7.17 | 15.7 | 9.79 | 1.09 | 0.63 | 1.92 | 0.17 | 0.54 | 1.36 |
| Area8 | 56.1 | 7.77 | 16.4 | 11.8 | 1.81 | 1.51 | 1.21 | 1.09 | 0.78 | 1.50 |
| Area9 | 54.1 | 7.19 | 17.6 | 12.0 | 1.72 | 1.74 | 2.30 | 1.10 | 0.8 | 1.46 |
| Area10 | 55.4 | 6.88 | 17.2 | 12.8 | 1.79 | 1.53 | 1.41 | 0.71 | 0.59 | 1.65 |
| Area11 | 56.8 | 8.65 | 16.1 | 11.9 | 1.26 | 1.22 | 1.45 | 0.68 | 0.51 | 1.50 |

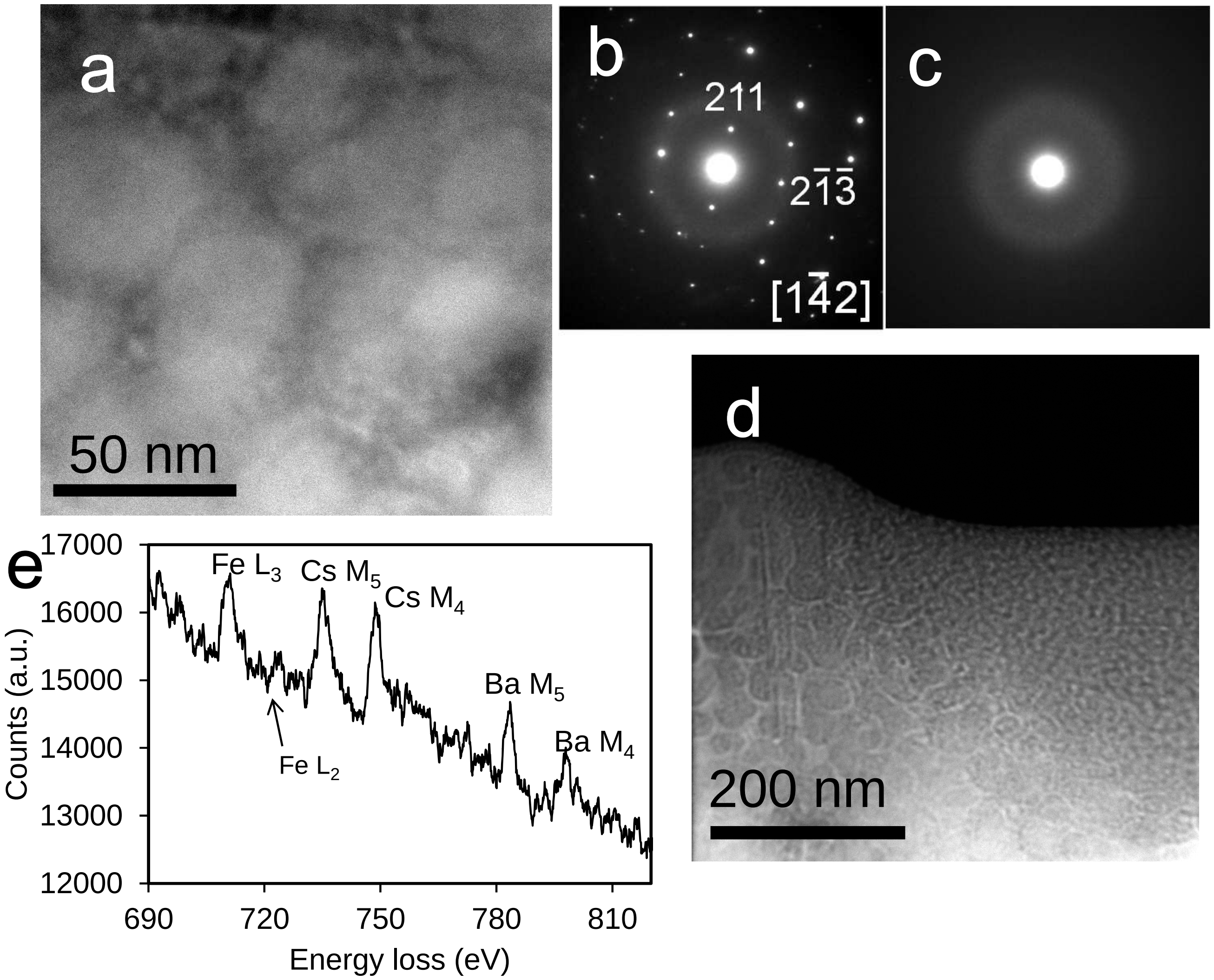


Figure S1. (a) HAADF-STEM image of the high Cs zone (represented by edx2 in Fig. 3b) in OTZ3-1 CsMP. (b) A SAED pattern of a large nanoparticle with high Cs content before amorphization by electron-beam irradiation. (c) A SAED pattern of a large nanoparticle with high Cs content after amorphization by electron-beam irradiation. (d) HAADF-STEM image of the boundary between high and low Cs zones (Fig. 3b) after amorphization by electron-beam irradiation. (e) Electron energy-loss spectroscopy of a high Cs zone.

Figure S1

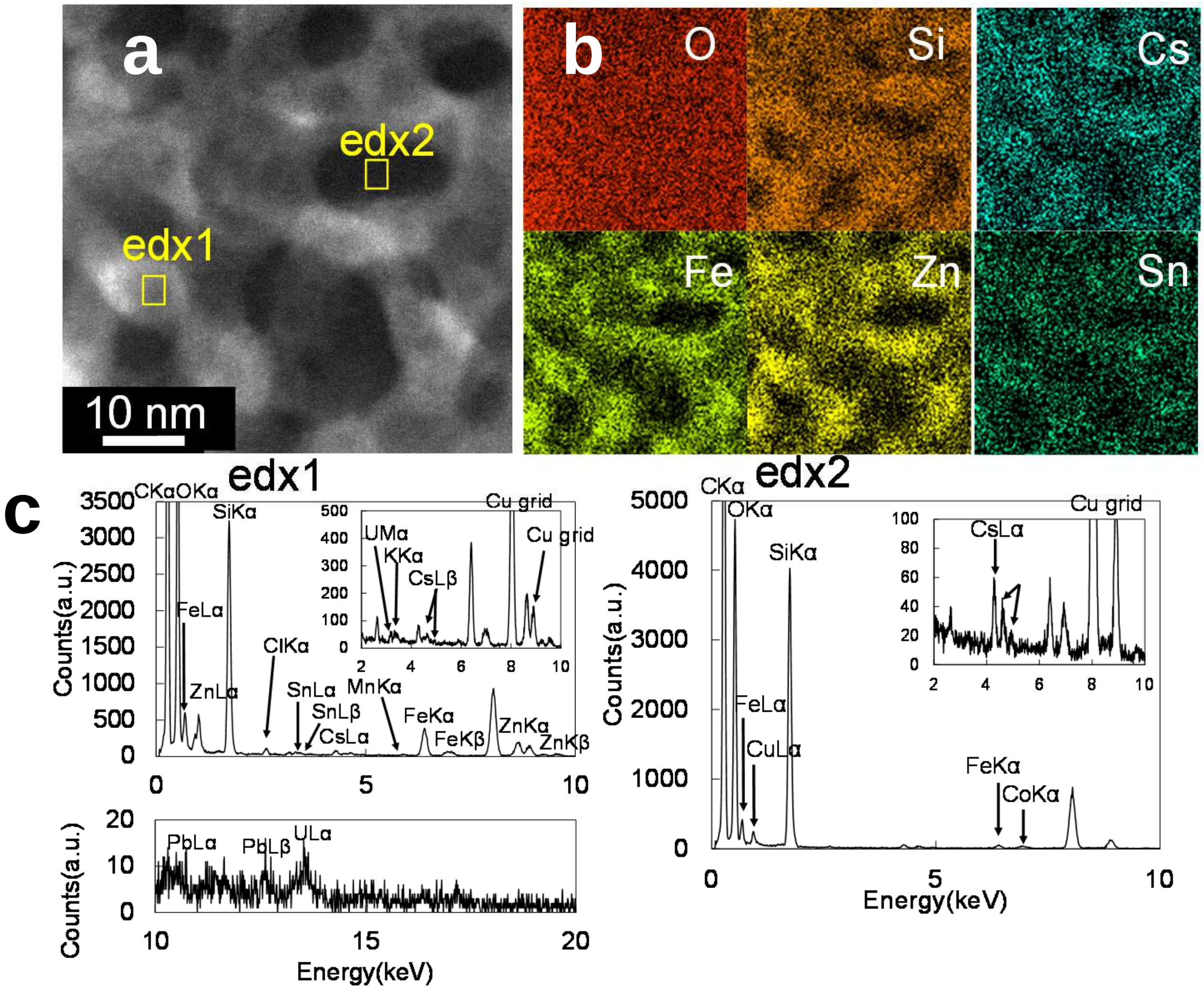


Figure S2. (a) HAADF-STEM image the OTZ10 CsMP. (b) Elemental maps of the major constituents for (a). (c) EDX spectra of the portion indicated by edx1 and edx2 in (a).

Figure S2
